# Health literacy reductionism: when individual deficits become the default explanation

**DOI:** 10.1093/heapro/daag118

**Published:** 2026-08-10

**Authors:** Tsuyoshi Okuhara, Hiroko Okada, Emi Furukawa

**Affiliations:** Department of Health Communication, School of Public Health, Graduate School of Medicine, The University of Tokyo, Tokyo 113-8655, Japan; Department of Health Communication, School of Public Health, Graduate School of Medicine, The University of Tokyo, Tokyo 113-8655, Japan; University Hospital Medical Information Network (UMIN) Center, The University of Tokyo Hospital, 7-3-1 Hongo, Bunkyo-ku, Tokyo 113-8655, Japan

**Keywords:** health literacy, health promotion, health communication, deficit framing, epistemic injustice, patient participation

## Abstract

Health literacy is one of the most influential concepts in contemporary health promotion. However, its success has created an explanatory shadow. Specifically, complex problems of communication, participation, and engagement are often reduced to deficits in individual health literacy, while relational, organizational, and structural explanations are backgrounded. This paper conceptualized this repetitive explanatory pattern as “health literacy reductionism,” the tendency to preferentially translate complex health communication and health promotion problems into a lack of individual health literacy among patients or the public. Drawing on and distinguishing this concept from adjacent critical discussions, including deficit framing, victim blaming, stigma, epistemic injustice, structural approaches, and medicalization, we argued that health literacy reductionism operates as a cross-cutting explanatory logic that can facilitate the reproduction of each of these phenomena. We presented a spectrum that distinguishes valid health literacy explanations from reductionist overextension, together with diagnostic questions for their identification. Four preliminary analytical codes (deficit attribution, presumed incomprehension, omitted alternatives, and design invisibilization) were proposed and applied to the published literature on medication adherence, clinical silence, shared decision-making, and vaccine hesitancy. This study highlighted the implications for research, health promotion, clinical practice, and institutional design.

Contribution to Health Promotion

- First, this paper provides a concept for identifying when low uptake, hesitancy, silence, or nonadherence might be framed too quickly as failures in individual understanding.
- Second, it offers preliminary analytical codes that can be applied to research papers, intervention materials, and policy documents to detect when programs default on education; however, structural, organizational, and trust-related barriers remain insufficiently examined.
- Third, it helps to redistribute explanatory accountability across individuals, institutions, and systems, thereby supporting a more reflexive design for communication, participation, and policy interventions in health promotion.

## Introduction: the success of health literacy and its explanatory shadow

Health literacy is an influential concept in contemporary health promotion and communications. Since Nutbeam (2000) positioned it as a key public health goal, health literacy has provided a powerful framework for highlighting the difficulties people face in accessing, understanding, evaluating, and utilizing health information and healthcare services (Nutbeam 2000, Pleasant and Kuruvilla 2008, Sørensen *et al*. 2012, Nutbeam and Lloyd 2021). Nutbeam (2000) distinguished three levels of health literacy (functional, interactive, and critical) to clarify that health literacy extends beyond basic reading and writing skills. More recently, this concept has been further expanded to encompass the responsibilities of organizations, systems, and environments that enable understanding and action (Trezona *et al*. 2017, Santana *et al*. 2021).

The success of this concept has cast an explanatory shadow on the understanding of health literacy. In both clinical and public health contexts, communication difficulties, nonparticipation, hesitancy, or health behaviors deemed inappropriate can too readily be read as consequences of “low health literacy.” For example, when patients do not follow a prescribed medication regimen (a behavior shaped by prescription complexity, cost barriers, side effect concerns, and trust in the prescriber), the explanation offered is often simply that these patients “lack adequate health literacy,” instead of examining the institutional and relational conditions that also contribute to nonadherence (Zhang *et al*. 2014, Jordão *et al*. 2025). As McCormack *et al*. (2017) argued, health literacy interventions should move beyond individual-level educational approaches and be understood within a broader social-ecological framework (McCormack *et al*. 2017). Osborne *et al*. (2022) warned that deficit-oriented interpretations of health literacy measurements can generate bias, stigma, and epistemic injustice (Osborne *et al*. 2022). Such explanations locate the cause within patients or the public, without further considering the significance of provider communication, institutional design, and unequal distribution of power and credibility (Viswanath and Kreuter 2007, McCormack *et al*. 2017, Osborne *et al*. 2022).

Notably, Nutbeam situated the social and political dimensions of health literacy through the concepts of interactive and critical health literacy (Nutbeam 2000). Nevertheless, despite these theoretical advances, functional and individually oriented dimensions remain especially prominent in health literacy measurements and empirical research (Osborne *et al*. 2022, Tavousi *et al*. 2022). Nutbeam and Muscat (2020) warned that “health literacy risks” are a convenient label for conventional health education interventions (Nutbeam and Muscat 2020). In a 25-year retrospective study, Nutbeam (2025) cautioned that health literacy should not be treated as a panacea for health inequities and called for stronger interventional research that can translate the concept into practical solutions (Nutbeam 2025).

Existing concepts have attempted to address these issues. Deficit-oriented interpretations of health literacy (Osborne *et al*. 2022), victim blaming (Jamrozik 2010), stigma (Parikh *et al*. 1996), epistemic injustice (Fricker 2007, Carel and Kidd 2014), and structural/organizational approaches (Brach *et al*. 2012, Bremer *et al*. 2021) have made crucial aspects, such as representation, responsibility displacement, devaluation, credibility undermining, and institutional conditions, more visible. The intent of this study is not to problematize these individual phenomena themselves, but rather the explanatory inertia: the tendency, once a given explanatory framework is established, for it to be repeatedly selected even when alternatives are available. Explanatory inertia is the means by which complex problems are repeatedly converged and explained as a lack of individual health literacy. Thus, a concept capable of grasping the explanatory logic that operates across these phenomena is required.

In this study, we conceptualized this pattern as “health literacy reductionism,” a recurring explanatory tendency that preferentially translates complex problems in health communication and health promotion into a lack of individual health literacy among patients or the public. This study aimed to address three key objectives: (i) conceptualize health literacy reductionism, (ii) distinguish this pattern from adjacent concepts and delineate boundary conditions, and (iii) demonstrate its analytical utility through application to published literature.

The health literacy reductionism lens draws attention to explanatory inertia and provides a tool for redirecting health promotion theory and practice toward a broader agenda, one that encompasses not only how individual understanding can be improved but also examines how explanatory accountability, institutional responsiveness, conditions of participation, and trust environments are distributed.

This concept applies across the full scope of health promotion, not only patient education; specific population-level instances (screening uptake, vaccine communication, and digital public health) are examined later in this paper. As a conceptual and interpretive analysis of previously published literature, this article involved no human participants, no identifiable personal data, and no animal subjects; ethical approval was therefore not required.

## Defining health literacy reductionism

### Definition, terminology, and definitional attributes

Health literacy reductionism refers to a recurring explanatory tendency that preferentially translates complex problems in health communication and promotion into a lack of individual health literacy among patients and the general public. However, the term “reductionism” does not denote an ontological or methodological position. Rather, it describes an explanatory contraction; despite multiple explanatory possibilities, individual attributes, such as understanding and knowledge, preferentially serve as the default explanation, while backgrounding other possibilities. Throughout this paper, we use “health literacy reductionism” to denote the explanatory pattern itself (specifically, the tendency to invoke individual health literacy, understood as a property of the person, as the default explanation), and “the health literacy reductionism lens” to denote the analytic perspective we propose for recognizing and addressing that pattern.

Why “reductionism?” The issue is not merely “spreading too wide” or “the first explanation that comes to mind,” but a movement in which relational, institutional, and political explanatory possibilities are excluded and contracting toward a narrow explanation of individual deficit. This study adopts “reductionism” as a term that signals contraction of explanation rather than an expansion of scope.

In this context, the usage of “reductionism” is not a strict transposition of philosophical reductionism but rather an alignment with critical usage in medicine and social sciences. Clark (2014) criticized the global health system’s tendency to insufficiently address social and political determinants, narrowly translating problems into medical solutions (Clark 2014). Wardrope (2015) argued that when the biomedical model is granted exclusive authority, as though it exhaustively describes all reality, social problems become individualized and depoliticized (Wardrope 2015). Rocca and Anjum (2020) criticized reductionism in the biomedical model as a tendency to reduce complex illnesses to lower physical and biological levels, obscuring the experience and context of the entire person (Rocca and Anjum 2020). In this study, the usage of “reductionism” aligns with this critical lineage. The aim is not to deny health literacy as a useful analytical resource but to highlight the explanatory contraction that occurs when one explanation is overused to explain the entire picture.

The definitional attributes of health literacy reductionism are three-fold. First, it reduces the complexity by translating multidimensional problems into presumed deficiencies in individual attributes. Second, it diverts attention from interactions, organizations, environments, and policy design and biases responsibility toward individuals. Third, it operates repeatedly across multiple research, practice, and policy sites, rather than as a one-off judgment.

### Conditions of occurrence

At least three conditions can be identified under which health literacy reductionism is likely to arise. First, the research culture prioritizes measurable individual attributes. Although Paasche-Orlow and Wolf’s (2007) causal pathway model positioned health literacy as a systemic phenomenon involving patient, provider, and system factors (Paasche-Orlow and Wolf 2007), much subsequent empirical research has preferentially tested the “patient factors” pathway while comparatively neglecting “system factors” and “provider factors” (Pleasant *et al*. 2016, Osborne *et al*. 2022). Second, the institutional environment is characterized by time pressure, increasing standardization of care processes, and persistent asymmetries in professional authority. In such settings, attributing a problem to a patient’s understanding may require less time and organizational effort than investigating provider communication, workflow design, or institutional barriers, making individual-deficit explanations the path of least resistance (Prasad *et al*. 2020, Sinsky *et al*. 2021, Stivers and Tate 2023). Third, the broader public discourse frames health outcomes primarily in terms of individual choice and behavior, even though health is shaped by commercial and structural determinants (Kriznik *et al*. 2018, de Lacy-Vawdon and Livingstone 2020, Mialon 2020). In such contexts, health literacy deficit becomes a readily available explanation for why individuals fail to make “the right choice.”

### Distinguishing valid explanations from reductionist overextension

This study does not deny the importance of health literacy itself. Rather, the purpose of this study is to distinguish between valid use and reductionist overextension. The concept is to be understood as a spectrum rather than a dichotomy. First, a valid health literacy explanation encompasses independent evidence of an individual’s difficulty in understanding or utilizing information, with alternative explanations considered, and where responsibility is not pushed unidirectionally onto the patient. For example, if persistent difficulties in understanding or utilizing specific information are confirmed even after providing simplified explanations, adequate time, interpreters, or supplementary materials, and assurance of a safe space for questioning, then a health literacy explanation may be valid. Second, the gray zone is when a situation or study is referenced to health literacy with some evidential basis, but consideration of alternative explanations is insufficient, or responsibility is beginning to tilt toward the individual. For instance, if an intervention study explains improvement in health outcomes solely through changes in health literacy scores with limited reference to the quality of information provided, the relational dynamics of the clinical setting, or institutional barriers, the study falls within the gray zone. Third, a clear reductionist overextension exists when the explanation leaps from observational findings to individual deficit, and interactional and institutional factors are scarcely considered, and responsibility is skewed one-directionally toward patients or the public.

The diagnostic questions used to identify this spectrum of health literacy reductionism are listed in Table 1. Table 1 is intended as a heuristic decision aid, and not a formal scoring instrument. Its purpose is to guide interpretive judgment by making explicit the dimensions along which valid explanations and reductionist overextensions can be distinguished. When a text scores for the overextension of multiple questions simultaneously, this constitutes stronger evidence of health literacy reductionism than any single indicator alone.

**Table 1:** Diagnostic questions for identifying health literacy reductionism.

Diagnostic question	Valid HL explanation	Gray zone	Reductionist overextension
Q1. Is there independent evidence of comprehension difficulty?	Independently assessed and confirmed	Reliant on inferences from HL scores	Inferred directly from observations without independent evidence
Q2. Have alternative explanations been considered?	Multiple alternatives substantively considered	Alternatives mentioned but consideration limited	Alternatives scarcely considered
Q3. Is there an explanatory leap from observations to individual deficit?	Relationship carefully examined	Tendency toward leap, with partial qualification	Observations immediately read as individual deficit
Q4. Is responsibility skewed toward patients/the public?	Distributed in multiple directions	Gradually tilting toward the individual	Almost entirely skewed toward patients/the public

## Positioning health literacy reductionism among adjacent critical discussions

Using this health literacy reductionism spectrum, we proceed to clarify how it relates to adjacent critical discussions. Each adjacent concept illuminates a specific phenomenon: how deficits are represented, how responsibility is displaced, how credibility is undermined, or how institutional conditions shape outcomes. None of them fully captures the repeated pattern by which these diverse phenomena converge to a single explanatory destination of individual health literacy. Health literacy reductionism names and analyzes this convergence. These distinctions are summarized in Table 2.

**Table 2:** Health literacy reductionism and adjacent concepts: a comparison.

Concept	Unit of analysis	Asks what?	Primary mechanism	What it misses
Deficit framing	Representation	How is individual deficit portrayed?	Representational practices	Why this representation was chosen over alternatives
Victim blaming	Moral attribution	Who is held responsible?	Responsibility displacement	The explanatory logic that enables the displacement
Stigma	Devalued identity	How does devaluation shape behavior?	Internalized shame and concealment	How stigmatized responses are re-read as deficit
Epistemic injustice	Credibility	Whose testimony is believed?	Prejudicial credibility deficit	Why HL deficit is preferred over credibility analysis
Structural approaches	Institutions/systems	What conditions shape outcomes?	Institutional design and responsiveness	Why individual-deficit explanations persist despite structural awareness
Medicalization	Category boundaries	How do conditions become medical problems?	Category creation and expansion	Excessive application of an already existing category
Health literacy reductionism	Explanatory logic	Why does this explanation recur?	Preferential explanatory convergence	— (cross-cutting)

To illustrate, a systematic review concludes that “low health literacy is significantly associated with medication nonadherence.” Considering the different perspectives, deficit framing would analyze how this statement represents an individual deficit (McCormack *et al*. 2017, Osborne *et al*. 2022), victim blaming would explore how it shifts moral responsibility onto the patient (Jamrozik 2010) and epistemic injustice would focus on how it undermines the patient’s credibility as someone who may have legitimate reasons for not adhering (Fricker 2007, Carel and Kidd 2014). In contrast, health literacy reductionism uniquely aims to illuminate why alternative explanations might not have been considered and why the patient’s health literacy was preferentially selected as the most natural explanation. When this contraction occurs repeatedly across systematic reviews, policy documents, and intervention designs, it becomes analytically independent. Given that structural and organizational perspectives are now well articulated, the distinctive question the health literacy reductionism lens asks is why explanations in practice and policy so often revert to individual deficit as the sole explanation.

That a thin conception of health literacy is the one most often operative is not incidental. Although Nutbeam’s (2000) model encompassed interactive and critical health literacy, measurement and intervention have remained predominantly functional: large-scale reviews rely on instruments such as TOFHLA and REALM (Berkman *et al*. 2011), and among 162 instruments developed between 1993 and 2021, functional measures dominated (Tavousi *et al*. 2022). When interventions stay centered on information provision, attention to upstream determinants drifts back toward individual responsibility, a “lifestyle drift” (Popay *et al*. 2010, Nutbeam and Lloyd 2021). Health literacy reductionism thus rests not only on the availability of health literacy explanations but on a thin, functional, and individual conception of the construct. Where richer conceptions (interactive and critical health literacy, and organizational and system responsiveness) are taken seriously, the reductionist move is itself disrupted, because these conceptions already locate part of the explanation in communicative, institutional, and systemic conditions. Invoking the fuller, multidimensional understanding of health literacy is therefore itself a safeguard against reductionism, and in this sense health literacy reductionism is not a rejection of the Nutbeam model but an adjunctive lens for analyzing why its goals so often remain practically unrealized.

## Preliminary analytical codes and their illustrative application

### Four analytical codes

To identify health literacy reductionism in research and practice, we proposed four preliminary analytical codes, which can be used in discourse and policy document analysis. These codes are intended as initial analytical scaffolds. Their reliability and validity require future systematic testing by independent coders. The four codes are deficit attribution, presumed incomprehension, omitted alternatives, and design invisibilization.

The four codes were derived conceptually rather than induced from a coded corpus. They follow from three sources: the three definitional attributes of health literacy reductionism (complexity reduction, responsibility diversion, and recurrence); the temporal structure of attribution, which distinguishes an *a priori* expectation (presumed incomprehension) from an ex post attribution (deficit attribution); and the analytic residue left by adjacent concepts, where omitted alternatives and design invisibilization name what deficit framing, victim blaming, epistemic injustice, and structural approaches each leave unaddressed. The codes are therefore preliminary and deductive, and establishing their reliability and validity requires future systematic application by independent coders.

“Deficit attribution” refers to the use of language that places the primary cause of an observed problem on an individual’s lack of knowledge, understanding, or judgment. For example, when medication nonadherence results from a patient’s inability to understand prescription instructions, this code is present. It captures the dimension of *ex post* attribution, that is, attributing cause after a problem has been observed.

“Presumed incomprehension” refers to a discourse that anticipates, without sufficient independent evidence that the person will not understand. This anticipation narrows the scope of explanation, or participation opportunities in advance. For example, this code is present when a clinician simplifies or withholds treatment options based on the assumption of the patient’s comprehension level, rather than an actual assessment. Whereas deficit attribution represents *ex post* attribution, presumed incomprehension represents an *a priori* assumption.

“Omitted alternatives” refer to cases in which plausible alternative explanations, such as those pertaining to interactions, organizational design, discrimination experiences, trust environments, or information infrastructure, are not considered or marginalized. For example, when a systematic review attributes nonadherence to low health literacy without examining other factors, such as cost barriers, provider communication quality, or prescription complexity, the code was present.

“Design invisibilization” refers to a discourse in which the design of tools, formats, or information environments is treated as neutral background conditions, so that difficulties arising from design choices are misattributed to individual deficiencies. For example, when a patient decision aid presupposes immediate verbalization and cognitive stability, and a patient who does not meet these assumptions is described as lacking ability. In this scenario, design invisibilization is a contributing factor.

### Approach and scope

The following illustrations are purposefully selected from the literature to represent different domains in which health literacy reductionism may operate. These are illustrative conceptual readings, not systematic reviews. The selections are used to demonstrate how the codes can be applied, and not how prevalent health literacy reductionism is across the field. The examples do not all represent explicit instances to the same degree. Some draw on published texts that explicitly attribute problems to health literacy deficits, while others illustrate domains where reductionist interpretation is more structural and implicit.

### Medication adherence in systematic reviews

Zhang *et al*. (2014) found a statistically significant but very small positive association between health literacy and medication adherence, with correlation coefficients below *r* = 0.10 across analyses. Despite this weak effect size, the review retained health literacy as the organizing construct, proposing that it functions as a “mediator” in relation to other adherence determinants. The review itself acknowledged that health literacy’s solitary impact is likely limited and named medication beliefs, provider-patient communication, and cost as relevant; yet by framing the analysis around health literacy, it illustrates how the construct’s operational readiness keeps it central even when the direct evidence is thin.

A more pronounced instance appears in Jordão *et al*. (2025), whose systematic review reported that having low health literacy increased the risk of unintentional nonadherence 2.6-fold. The authors concluded that health literacy screening should be integrated into polypharmacy management protocols, foregrounding individual health literacy screening as the gateway response, without elaborating on the institutional and relational factors. The two reviews thus occupy different points on the spectrum introduced above: Zhang *et al*. approaches the restrained pole, naming multiple determinants and cautioning against over attribution, yet still organizes explanation around health literacy, whereas Jordão *et al*. moves toward reductionist overextension by converting a single association into an individual-screening recommendation.

These reviews exercise the codes to differing degrees. In Jordão *et al*., “deficit attribution” is present where low health literacy is framed as a “critical” and “modifiable” determinant and policy-makers are urged to prioritize health-literacy-sensitive interventions; “presumed incomprehension: operates in the recommendation of individual health literacy screening as the gateway response, which treats anticipated low comprehension as the operative cause before institutional and relational determinants are examined; and “omitted alternatives” is at work in that, although the review notes provider-side communication gaps (including the absence of any confirmation of patient understanding in most encounters) and recommends label and format redesign, its lead recommendation still defaults to individual screening, leaving institutional and relational factors analytically subordinate. “Design invisibilization” does not fire for these reviews, precisely because redesign of labels and formats is treated as a modifiable remedy rather than neutral background; that code is exercised instead in the shared decision-making example below. Zhang *et al*. sits at the restrained pole: it names beliefs, communication, and cost as determinants and cautions that health literacy’s solitary impact is limited, so the codes find little purchase beyond the residual pull of an operationally ready construct.

Read together, the codes locate the explanatory flip precisely: in Jordão *et al*., a single risk ratio (a 2.6-fold increase) is converted into a recommendation for individual screening, while in Zhang *et al*. an even weaker association (*r* < 0.10) is nonetheless retained within a health-literacy framing rather than relocated to other determinants; it is this conversion of modest, individual-level statistics into a person-level response that renders prescription design, communication quality, and cost barriers comparatively invisible.

By contrast, a description that avoids health literacy reductionism would read as: “A higher incidence of medication nonadherence was observed among patients with low health literacy scores. However, this association must be interpreted in light of the complexity of prescription information, time constraints, cost-related intentional nonadherence, and side effects. Strategies should include prescription design improvement, pharmacist follow-up, cost barrier reduction, and health literacy support.”

### Silence in clinical settings and professionals’ anticipatory judgments

When patients do not ask questions, this is sometimes read as a sign of limited health literacy; the four codes, however, direct attention to what such a reading can omit. Parikh *et al*. (1996) studied patients at public hospitals and found that many with low functional literacy experienced reading difficulties as a deep shame. Moreover, these difficulties may not be known to family members. Consequently, the patients avoid situations in which their limited literacy might be exposed, including asking questions. Through qualitative interviews with adults who experienced literacy difficulties, Easton *et al*. (2013) showed that stigma can lead to systematic question suppression and reduce the quality of spoken interactions. Silence is often an interactional defense against anticipated humiliation, and is not necessarily a sign of a deficit. Interpreting silence as a lack of understanding constitutes “deficit attribution.” If shame and stigma are not considered, “omitted alternatives” are also at work. If consultation time pressure and professional authority asymmetries are not examined, “design invisibilization” is further implicated. Furthermore, the consultation format itself may be treated as neutral rather than a potential source of the problem. Therefore, the focus of the intervention should not be limited to patient education. Instead, the focus should shift toward designing interactions that are less likely to generate shame, ensuring relational safety in which questions do not damage relationships, and fostering a clinical culture that does not link the expression of comprehension difficulties with devaluation.

Furthermore, Clinch and Benson (2013) found through interviews with 24 general practitioners in the United Kingdom that physicians routinely engaged in “preemptive work,” making advance judgments about what information was “relevant” to each patient and filtering explanations accordingly, often without verifying whether patients wanted such filtering (Clinch and Benson 2013). Here, “presumed incomprehension” is centrally at work: the patient’s comprehension difficulty is not “discovered” but “preemptively constructed.”

### Participation formats and shared decision-making

In the implementation of shared decision-making, difficulties in participation are easily interpreted as deficits in patient capacity. However, from the perspective of health literacy reductionism, the key question is whether the formats and conditions that facilitate participation are visible. When the participation timing, mode of decision aids, consultation workflow, and assumptions built into the tool are treated as neutral conditions, difficulties arising from them may erroneously be attributed to the patient. This represents “design invisibilization.”

Jones *et al*. (2009) found that the relative efficacy of a statin decision aid interacted with how it was delivered: knowledge gains were clearest when the clinician delivered it during the visit rather than a researcher beforehand, though several differences were modest and not statistically significant (Jones *et al*. 2009). Similarly, Scalia *et al*. (2018) found that the use of option grids depended not only on patient characteristics, but also on the clinical context and clinician engagement (Scalia *et al*. 2018). In that study, the same Option Grid produced significant improvement for only five of thirteen clinicians (all at a single clinic) and a significant decline for one, with outcomes tracking clinician attitude, training, and a local “champion” rather than patient capacity; the statin Option Grid did not even reproduce the benefit seen with the Mayo statin decision aid, underscoring that identical tools succeed or fail by their conditions of use. These studies suggest that before participation difficulties are interpreted as problems relating to patient ability, the format of participation itself must be examined. Friction in shared decision-making should not be read too quickly as evidence that “the patient cannot participate.” The patient may have been placed within a participatory format that is difficult to navigate.

In the terms of the four codes, reading this friction as evidence that the patient cannot participate is “deficit attribution”; filtering options or decision aids by an assumed level of capacity is “presumed incomprehension”; and the omission of delivery, clinical context, and clinician engagement (the very factors Jones *et al*. and Scalia *et al*. identify) is “omitted alternatives” operating alongside the “design invisibilization” already noted.

### Public communication and hesitancy

Health literacy reductionism occurs when public hesitancy is processed primarily as low individual health literacy while trust, discrimination, and commercial influences are backgrounded. Lorini *et al*. (2018) emphasized the importance of improving health literacy in addressing vaccine hesitancy in a systematic review. However, later studies suggest that hesitancy and mistrust cannot be explained by health literacy alone. Weerakoon *et al*. (2022) found that low trust in health professionals was strongly associated with COVID-19 vaccine hesitancy (Weerakoon *et al*. 2022), while Cénat *et al*. (2023) showed that vaccine mistrust was associated with racial discrimination in healthcare, as well as health literacy and conspiracy beliefs (Cénat *et al*. 2023, 2024). Here, “omitted alternatives” are most prominent when trust, discrimination, and misinformation environments are treated as secondary to information deficits.

A similar explanatory pattern can be observed in population-level health promotion beyond vaccination. When the low uptake of screening programs is interpreted primarily as a failure of public understanding, the policy response may default to intensifying information provision or literacy-focused education, even though screening participation is shaped by broader barriers, such as access, cost, provider recommendation, and social conditions (Lin *et al*. 2022, Aguiar-Ibáñez *et al*. 2025). Similar tendencies may also appear in preventive campaigns and digital public health tools, where nonengagement is read mainly as an informational problem rather than a lack of trust in institutions, discrepancies in communicative fit, or the design and engagement demands of digital interventions (Krastev *et al*. 2023, Milne-Ives *et al*. 2023, Lapinski *et al*. 2025). In such cases, “omitted alternatives” and “design invisibilization” can work together. The infrastructure of access and participation may remain under-examined, even though organizational health literacy research emphasizes the need to assess and improve service responsiveness (Farmanova *et al*. 2018, Trezona *et al*. 2018, Bremer *et al*. 2021). This is precisely why health literacy reductionism matters not only for clinical encounters but also for population-level health promotion.

### Potential for extension to quantitative research

These codes are applicable not only to qualitative text analysis but also to quantitative research design. For example, one could develop vignette experiments in which the same behavior is paired with different explanatory conditions, such as health literacy deficits, cost barriers, the lack of trust, or prescription design problems, and ask healthcare professionals or policy-makers to rate the plausibility, naturalness, and intervention priority of each explanation. Such designs would make it possible to examine whether health literacy explanations are systematically favored over structural or relational alternatives when the underlying scenario is held constant.

A systematic content analysis of published literature using these codes can also provide quantitative evidence of how frequently reductionist explanatory patterns appear within a given body of research, especially when combined with interrater reliability testing. Together, these approaches address different aspects of the problem. Content analysis can identify the prevalence and distribution of explanatory patterns in the existing literature, whereas vignette experiments can test explanatory preferences under controlled conditions. At the same time, identifying the broader conditions under which health literacy explanations become disproportionately favored will likely require attention to the institutional incentives, funding structures, and practical constraints that make individual-level explanations more readily operationalized than systemic ones. Therefore, an empirical study of health literacy reductionism will likely require sustained multimethod development.

## Implications: suggestions for research, practice, and institutional design

### Implications for research

Studies using health literacy as an independent variable remain important. However, researchers should examine whether their explanations converge on individual deficits, while marginalizing structural and relational factors. These codes can be applied to research papers, guidelines, policy documents, and interventions. Researchers can assess reductionist tendencies by examining whether a text primarily attributes a problem to a patient’s understanding, or considers institutional design, communication quality, cost barriers, and trust. When multidimensional measurement tools, such as the HLQ or HLS19 are used, researchers should examine whether the results are ultimately channeled into individual-level intervention proposals or whether they inform systemic responses.

### Implications for practice

Safeguards should be built into clinical practice to prevent silence, nonquestioning, and nonparticipation from being immediately read as deficit signs. When communication efforts do not produce the expected responses, clinicians should systematically re-examine the mode of explanation, time allocation, relational safety, and terminology, rather than defaulting to the assumption that the patient lacks understanding. Training modules in clinical education should teach interpreting silence not as an indicator of ability but as a potential indicator of interactional safety deficits. In evaluating shared decision-making tools, the criteria should include whether hesitation and objection are also legitimate forms of participation.

### Implications for institutional design and policy

Organizational health literacy responsiveness should not be limited to plain language provision, but should also address how services are structured, accessed, and experienced (Brach *et al*. 2012, Koh *et al*. 2013; Trezona *et al*. 2017, 2018). Policy documents should be reviewed to assess the attribution of responsibilities, considering individual deficits, as well as systemic conditions. Such a review would ask whether poor outcomes are framed mainly as consequences of individual understanding or whether institutional design, communication quality, and trust environments are also examined. Stakeholder perspectives should be incorporated from the study design stage through community codesign approaches, such as Ophelia (Beauchamp *et al*. 2017). This is particularly important in population-level health promotion, where responses to low uptake or weak engagement may otherwise default to education provision, while barriers to access, trust, and participation remain insufficiently addressed.

### Responses to anticipated counterarguments

First, health literacy reductionism is merely a renaming of existing critiques. However, its object is not an individual phenomenon, but a cross-cutting explanatory logic that facilitates their reproduction, as Table 2 demonstrates. Health literacy reductionism is, we acknowledge, a species of the broader explanatory reductionism long criticized in public health. Its contribution is one of diagnostic specificity: health literacy is among the most operationalized constructs in the field, carrying its own measurement instruments, intervention paradigms, and policy uptake, and this operational readiness is precisely what gives the reductionist pull its strength and renders alternative explanations comparatively invisible. A general injunction against reductionism cannot be applied to a specific body of literature, but the four codes can, and they are health-literacy-specific. Second, while low health literacy is sometimes the primary factor, this study aimed to demonstrate the boundary conditions. Third, multidimensional measurement tools can be used to resolve this problem. However, as discussed earlier, multidimensional measurement does not structurally prevent reductionism at the level of explanatory logic.

### Limitations

This study is a conceptual and interpretive essay. The cases analyzed in earlier sections are illustrative and not comprehensive. While they demonstrate how the four analytical codes can be applied, it was not possible to establish the prevalence of health literacy reductionism across the field. The analytical codes have been proposed as a preliminary scaffold, and evaluating their reliability and validity requires future systematic application by multiple independent coders. Additionally, this study relied primarily on Western and Anglophone literature. Hence, the concept’s applicability to different healthcare systems and cultural norms requires future comparative examination.

## Conclusion

This paper proposed “health literacy reductionism” as a concept that focuses on unpacking the recurring and recognizable explanatory tendency for complex problems of communication and participation to be preferentially translated into deficits of individual health literacy, while relational, institutional, and political explanatory possibilities are inadequately considered. The concept is applicable across research, practice, and policy. To illustrate health literacy reductionism as an analytically concrete concept, we defined four preliminary analytical codes and demonstrated their application to existing literature. The systematic empirical validation of health literacy reductionism remains a task for future research.

The deeper issue this concept illuminates is not that health literacy explanations are wrong, but that they are too readily available, too natural, too measurable, and too actionable, compared to explanations that implicate institutions, trust environments, or information ecologies. This asymmetry in explanatory ease makes health literacy reductionism self-reinforcing: the more it is used, the more natural it appears, and the less visible the alternatives become. What health promotion needs, therefore, is not less attention to health literacy, but disciplined vigilance about what becomes invisible each time health literacy is invoked as an explanation. As a heuristic concept, health literacy reductionism is offered not to displace health literacy research but to improve the explanatory discipline with which health promotion problems are framed, studied, and acted upon.

## Data Availability

Data sharing is not applicable to this study because no datasets were generated or analyzed.
